# Complete Genome Sequence of a Quorum-Sensing Bacterium, *Oceanicola* sp. Strain D3, Isolated from a Microplastic Surface in Coastal Water of Qingdao, China

**DOI:** 10.1128/MRA.01022-19

**Published:** 2019-10-03

**Authors:** Qian Li, Xiyuan Xu, Changfei He, Li Zheng, Wei Gao, Chengjun Sun, Jingxi Li, Fenglei Gao

**Affiliations:** a Key Laboratory for Marine Bioactive Substances and Modern Analytical Technology, First Institute of Oceanography, Ministry of Natural Resources, Qingdao, China; b Laboratory for Marine Ecology and Environmental Science, Pilot National Laboratory for Marine Science and Technology (Qingdao), Qingdao, China; c Laboratory of Marine Drugs and Bioproducts, Pilot National Laboratory for Marine Science and Technology (Qingdao), Qingdao, China; Georgia Institute of Technology

## Abstract

Oceanicola sp. strain D3 was isolated from the plastisphere of polyvinyl chloride (PVC) in coastal water of Qingdao, China. Here, we present the complete genome sequence of strain D3, which consists of a chromosome of 3,926,685 bp with a G+C content of 64.49% and 4,964 coding DNA sequences. This is the first report of a quorum-sensing (QS) system in an *Oceanicola* sp. strain.

## ANNOUNCEMENT

Microplastics have emerged as new pollutants in oceans (1, 2). Their fates, including migration and degradation in marine environments, have become research hotspots, and evidence shows that microorganisms associated with microplastics play a key role in the processes (3). Quorum-sensing (QS) bacteria have been discovered to be prevalent microorganisms on the surfaces of microplastics (4). QS is a cell-cell communication system in bacteria through sensing of the density of *N*-acyl homoserine lactone (AHL) signals (5). Many important ecological functions of marine QS bacteria, such as bioluminescence, biofilm formation, algicidal activity, and settlement of seaweed zoospores and invertebrate larvae, are regulated by the QS system (6–8). However, the role of QS bacteria on marine microplastics is still unknown.

**FIG 1:**
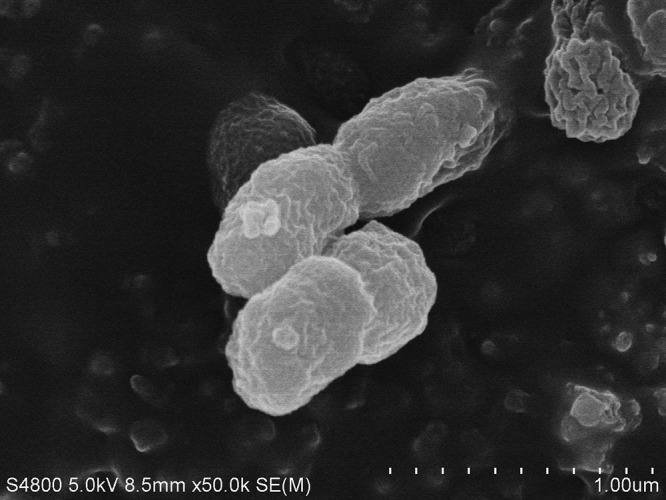
Scanning electron microscope image of *Oceanicola* sp. strain D3.

The Oceanicola genus belongs to the family Rhodobacteraceae in the phylum Alphaproteobacteria, which was first described by Sooyeon Park (9). In this genus, only one strain, Oceanicola litoreus M-M22, isolated from seashore sediment, was previously identified (9). Our strain, *Oceanicola* sp. strain D3, was isolated from the plastisphere of polyvinyl chloride (PVC) in coastal water of Qingdao, China. PVC plastic particles were put into sterile water under oscillation cleaning in order to obtain the bacteria from the plastisphere. The strain was incubated on a 2216E plate (10) at 25°C for 48 to 72 h, and then the unique colonies were obtained. *Oceanicola* sp. strain D3 is a long rod-shaped aerobic bacterium, approximately 0.3 to 0.5 μm wide and 0.6 to 1.0 μm long (Fig. 1 A). Here, we performed complete genome sequencing of strain D3. Using 16S rRNA gene sequence analysis and a BLAST search against the NCBI database, we found that the most similar strain is the type strain, *Oceanicola litoreus* M-M22, with a similarity of 98%.

Genomic DNA of strain D3 was extracted with a PowerSoil DNA isolation kit (MoBio, Carlsbad, CA) on the basis of the manufacturer’s instructions. The DNA sample was sheared, using a Covaris g-Tube, into the desired size fragment (10 kb) of the library. After DNA damage repair, the hairpin-type adaptor was ligated to both ends of the DNA fragment using DNA T4 ligase. The 10-kb SMRTbell library was constructed with purified DNA fragments by AMPure PB magnetic beads (11). The constructed library was quantified by Qubit quantitation, and the insert size was detected using an Agilent 2100 instrument (12). PacBio sequencing was performed at the Beijing Novogene Bioinformatics Technology Co., Ltd. Raw data were filtered to obtain clean data (reads, >500 bp; read quality, >0.75). A total of 238,228 reads with an average length of 8,110 bp were obtained for a total of 1,932,078,583 bp of the sequence. *De novo* assembly was done using the Hierarchical Genome Assembly Process (HGAP) workflow implemented in the single-molecule real-time (SMRT) analysis software SMRT Link v5.0.1 (13, 14). The genome sequence was annotated and analyzed using GeneMarkS version 4.17 (http://topaz.gatech.edu/GeneMark/) (15). tRNA operons were assessed via tRNAscan-SE version 1.3.1 (16), and rRNA operons were assessed by RNAmmer version 1.2 (17). Default parameters were used for all software unless otherwise noted.

The genome sequence of strain D3 was assembled into one contiguous scaffold with 450-fold coverage, which represents a single circular 3,926,685 bp chromosome with a G+C content of 64.49%. The genome is predicted to contain 4,405 coding sequences. The numbers of tRNAs and rRNA operons are 44 and 1, respectively. In a typical AHL-QS system, *luxI* is the AHL synthase gene and *luxR* is a modular transcriptional response regulator gene (18). From the chromosome of strain D3, one cluster of *luxI* and *luxR* homologues was identified, and the *luxR* homologue is located in the upstream position. The genes of *luxI* and *luxR* homologues are located at Chr1:1171346:1171984 and Chr1:1170512:1171228, respectively.

### Data availability.

The complete genome sequence of *Oceanicola* sp. strain D3 has been deposited at DDBJ/EMBL/GenBank under accession number CP040932 and BioProject number PRJNA547459. The raw sequencing reads have also been submitted to the Sequence Read Archive (SRA) under accession number SRR9875591.
